# Sustainable Use of *Macrotermes* spp. to Improve Traditional Poultry Farming through an Efficient Trapping System in Burkina Faso

**DOI:** 10.3390/insects13010062

**Published:** 2022-01-05

**Authors:** Aïchatou Nadia Christelle Dao, Fernand Sankara, Salimata Pousga, Kalifa Coulibaly, Jacques Philippe Nacoulma, Irénée Somda, Marc Kenis

**Affiliations:** 1 Institut du Développement Rural (IDR), Université Nazi Boni (UNB), Bobo-Dioulasso 01 BP 1091, Burkina Faso; christelledao@yahoo.fr (A.N.C.D.); ferdisank2005@yahoo.fr (F.S.); pousgasalimata@yahoo.fr (S.P.); coulkal1@gmail.com (K.C.); nacphil2@yahoo.fr (J.P.N.); ireneesomda@yahoo.fr (I.S.); 2 CABI, 1 Rue des Grillons, 2800 Delemont, Switzerland

**Keywords:** Burkina Faso, feed, *Macrotermes*, poultry farming, substrates, termites

## Abstract

**Simple Summary:**

In West Africa, termites are commonly used as protein feed for poultry by smallholder farmers who trap them by placing containers filled with organic matters on termite tracks. The objective of this study conducted in Burkina Faso was to test and improve the technique to increase the availability of termites for traditional poultry farmers. The study focused on termites of the genus *Macrotermes* and found that the quantity of termites trapped varied with the containers’ types, substrates placed in the container, habitats, harvesting times, duration of trap deposition, and season. These results allow to provide information to farmers on how to optimize the trapping of *Macrotermes* species and increase the quantity of termites available as protein source for their poultry.

**Abstract:**

This study aimed to promote the use of termites as feed in traditional poultry farming by developing sustainable and inexpensive termite trapping techniques. Various tests were carried out in Burkina Faso to improve the traditional technique of trapping termites of the genus *Macrotermes* using a reversed container filled with organic matters. We studied the effect of containers’ types, substrates, habitats, harvesting times, duration of trap deposition, and season on the quantity of termites trapped. Calabashes and terra cotta pots trapped more termites than iron boxes, but calabashes were quickly destroyed by termites. The quantity of termites harvested increased proportionally with the volume of the pot and was higher in a cultivated habitat than in a forest, despite the higher number of termite mounds in the forest. The quantity of termites harvested was lowest in the cold-dry season and highest during the rainy season, however, sizeable amounts of termites were collected throughout the year. Among six substrates and mixtures of substrates tested, maize cobs trapped the highest number of termites and cow dung the lowest. The best time of harvest varied among seasons and, if substrates are abundant, it is more efficient to empty the containers on a daily basis.

## 1. Introduction

Termites play an important role in tropical ecosystems. They are major agents in the preliminary stages of the decomposition of plant litter, particularly in savannas and arid forests of Africa [1]. In addition, termites are rich in protein and are commonly used as human food and animal feed. They are the second most consumed insect order after Orthoptera and are among the most nutritious insects [2]. Termites can be easily collected around farms at low cost [3]. By using termites, rural poultry farmers can offset the high cost of animal protein (e.g., fish meal) and plant protein (e.g., cottonseed cake and soybean meal) used in animal feed, in particular poultry and fish. The use of termites in food and feed has already been described by many authors e.g., [4,5,6,7,8,9]. In West African countries such as Togo, Ghana, Benin and Burkina Faso, termites are traditionally used to feed poultry in smallholder farms [7,8,10,11,12].

In Burkina Faso, the main termite genera used in poultry feed by rural poultry farmers are *Macrotermes*, *Trinervitermes*, *Cubitermes*, and *Odontotermes* [8,13]. Studies by Pousga et al. [3] have shown that the termite species *Macrotermes subhyalinus* (Rambur) and *Macrotermes bellicosus* (Smeathman) could theoretically substitute imported fishmeal in growing rations for poultry. Ideally, 10% of the chicken diet should be supplied as termites or other insects. Different harvesting techniques are used for different termite genera. Termites of the genus *Macrotermes* are mainly harvested in two ways: By partial destruction of the termite mound and by trapping [7,8]. Some poultry farmers have developed controlled termite trapping techniques that preserve termite mounds [6,7,8]. In Burkina Faso, these techniques are mainly based on used containers such as terra cotta pots, iron or plastic containers, calabashes, or baskets made from leaves of palmyra palm *(Borassus akeasii)*. These containers are filled with plant and animal substrates, such as crop residues, tree debris, and animal dung [7,8,14]. However, techniques for trapping or collecting termites are not well mastered by all poultry farmers. In addition, during certain periods of the year, it is more difficult to obtain termites than in other periods and the quantities obtained are often small. This makes termite collection difficult for smallholder farmers, who often decide to abandon the method [12]. In view of the high nutritional quality of termites, their availability in the natural environment and their benefits on poultry growth, there is a need for a better understanding of termite trapping techniques in order to improve them. This will allow efficient and sustainable use of these insects in poultry feed and facilitate the work of poultry farmers. This study was conducted to evaluate the effect of factors that can influence the quantity of termites of the genus *Macrotermes* trapped according to the season. Specifically, the aim of the study was to: (i) Identify the best containers and substrates for *Macrotermes* spp. trapping; (ii) evaluate the effect of trap sites and the duration of trap deposition on the quantity of *Macrotermes* spp. harvested according to the season; and (iii) identify the best times of the day for *Macrotermes* spp. harvesting according to the season.

## 2. Materials and Methods

### 2.1. Experimentation Area

**Figure 1:**
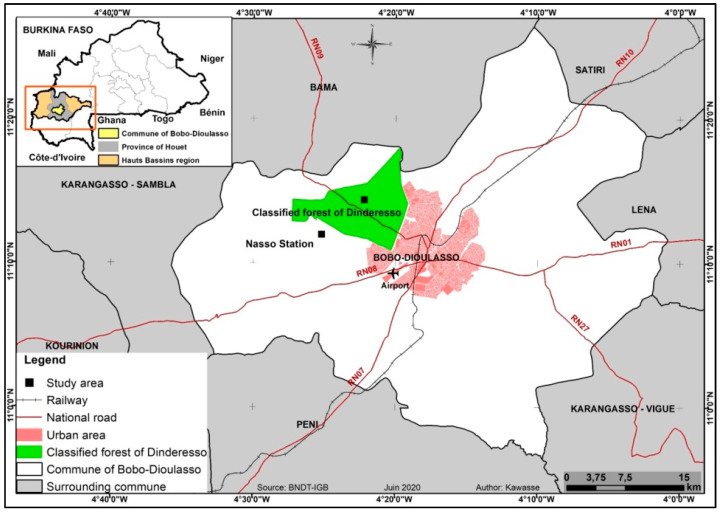
Location of the experimental sites. Source: National Topographic Database-Geographic Institute of Burkina Faso.

**Figure 2:**
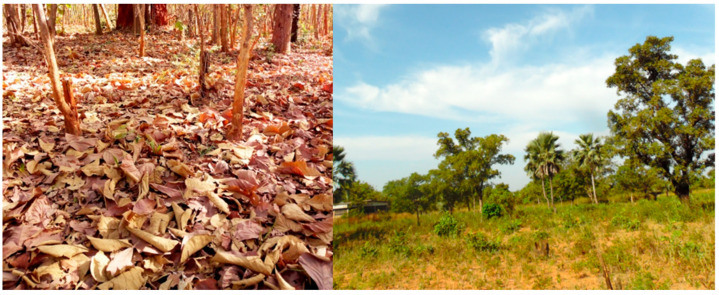
View of the two sites: Dinderesso (**left**) and Nasso (**right**).

Termite trapping tests were carried out in the Hauts-Bassins region in Burkina Faso, about 15 km north-west of the city of Bobo-Dioulasso, in the Nazi Boni University (UNB) property in the village of Nasso (4°41′ W; 11°20′ N) and in a classified forest in the village of Dinderesso (4°43′ W; 11°22′ N) (Figure 1). The region is located in the southern Sudanese zone, where the rainy season lasts 5 to 6 months with 1100 mm precipitation per year [15]. The Nasso station is located in a woody savannah where vegetation is mainly composed of *Detarium microcarpum* G., *Daniellia oliveri* H. and D., *Vitellaria paradoxa* C. F. and G., *Parkia biglobosa* J., *Borassus* spp., *Eucalyptus* spp., Poaceae, etc. [16]. At the Dinderesso site, the dominant species were large trees of *Tectona grandis* L. Other trees included *Daniellia oliveri* (Rolfe) Hutch. and Dalz, *Nauclea latifolia* Smith, *Guiera senegalensis* J.F. Gmel, *Cola cordifolia* (Cav.) R. Br., *Pilliostigma thonningii* (Schumach.) Milne-Redh, and *Khaya senegalensis* (Desr.) A. Juss. The soil was mainly covered by Poaceae. At Dinderesso, the soil was covered by a thick litter composed of tree leaves whereas the litter was much thinner at Nasso (Figure 2).

### 2.2. Identifying Trap Deposition Sites

To ensure the colonization of a substrate (food bait) by termites, it is necessary to verify their presence on the site. This requires attracting them first before setting up a device. This step consisted, firstly, in identifying the presence of plant or animal matters (e.g., animal droppings) attacked by termites on the ground; secondly, in depositing a small quantity of substrate (about 100 g) on the mud tubes indicating the termites’ tracks on the surface of the ground, at a distance not exceeding 1.5 m from one point to another. During the rainy season, 100 g of substrate was covered by terra cotta pots to protect them from the rain. Twenty-four hours later, if the substrate was colonized by *Macrotermes* spp., the site was considered suitable. If, on the other hand, 24 h later, the substrate was not colonized by termites or colonized by other species, the site was not considered for the study. Preliminary observations had shown that, at the two sites, two species of *Macrotermes* are present, *M. bellicosus* and *M. subhyalinus*, the second one being the most abundant. However, during this study, we did not attempt to identify the *Macrotermes* species in each trap.

### 2.3. Trapping Method

**Figure 3:**
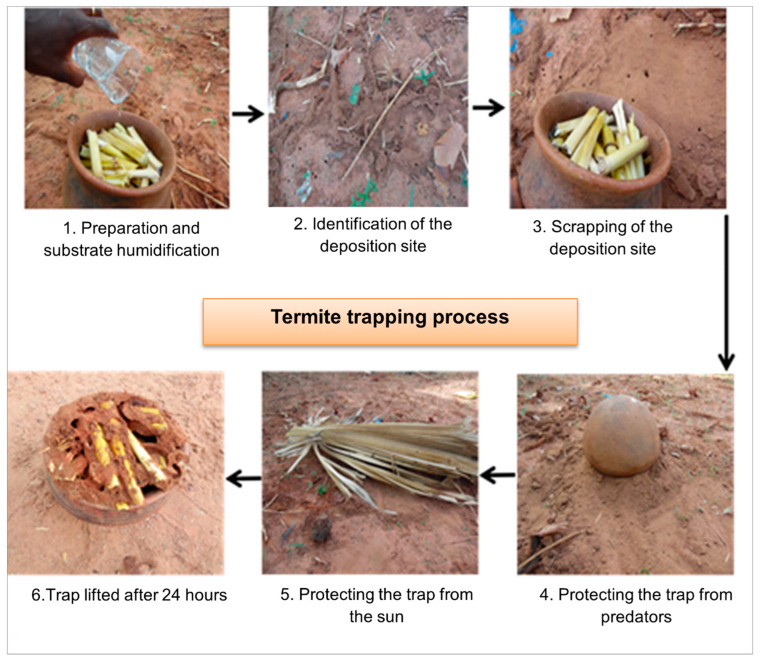
Termite trapping process.

Termite harvesting was carried out following the method practiced by poultry farmers on farms, based on containers filled with substrates. This method has been described by many authors e.g., [7,8,11]. The general procedure was as follows: Substrates were placed in the container and moistened with water; the reversed container was placed on scraped soil at a site previously selected as suitable for termite trapping; the trap was protected from predators by closing the borders of the container with soil and from sunlight by covering it with leaves of palmyra palm; at the end of the trapping period, the trap was lifted and emptied. The process is illustrated in Figure 3.

### 2.4. Harvesting, Drying, and Weighing of Termites

Harvesting consisted of emptying the container with the colonized substrate and sorting out the termites (Supplementary File S1 Figure S1). First, the content was poured into another container and the large particles of substrate not consumed by the termites were sorted by hand. Then, the rest of the content, consisting of soil, and small pieces of substrate and termites, was placed on stacked sieves with mesh sizes ranging from 0.2 cm to 2 cm with the larger mesh placed above. After sieving the substrate, only a little bit of sand and termites remained on the last sieve, which was placed in a container with water. After stirring, the rest of the sand went down into the water, leaving the termites and some impurities smaller than 0.2 cm on the sieve. The termites were rinsed in two to three containers of water until they were clean. The last pieces of substrates were removed with soft tweezers. After sorting, the termites were placed into plastic containers and left to dry in the sun for 24 h in the dry season and 48 h in the rainy season. While drying the termites at a constant temperature in an oven would have provided more consistent data throughout the year, this option was not available on site. After drying, the termites were placed into labeled plastic bags and weighed in the laboratory using a 0.01-g precision balance. The processes described above were applied to all the experiments carried out during the study. The variables measured during the experiments were the masses of termites dried in the sun, the temperature, and the relative humidity of the environment during harvesting. All data on average temperature and relative humidity during harvest are shown in the Supplementary File S1, Tables S1 and S2.

### 2.5. Influence of Containers on Termites Trapping

To test the effect of the containers on termite trapping, an experiment was carried out at the Nasso station and in the classified forest of Dinderesso in November 2017. Three types of containers were used: Terra cotta pots, iron boxes, and calabashes (fruits of *Lagenaria* sp.). Containers with a volume of 2 dm^3^ and an opening of 15.5 cm in diameter for the iron container, 15.5–16.5 cm for the terra cotta pots, and 17 cm for the calabash were used. For the experiment, a completely randomized block design (blocks dispersed in time) with three treatments (containers) and five replicates per day was carried out over four days, i.e., 20 repetitions for each treatment. The blocks were considered as successive trials [17,18,19,20].

### 2.6. Influence of Seasons and Substrates on Termite Trapping

Experiments were carried out at the Nasso station to evaluate the quantities of termites trapped according to substrates and seasons. Substrates were chosen based on the results of surveys conducted on termite use in Burkina Faso [8] and the results of our preliminary tests on station. Three substrates (cow dung, maize cobs, and maize stems) were used to form six treatments (maize cobs (Ms), maize stems (MS), cow dung (CD), maize cobs and stems mix (Ms + MS), maize cobs and cow dung mix (Ms + CD), and maize stems and cow dung mix (MS + CD)). Terra cotta pots (volume: 2 dm^3^, diameter of the opening: 16 cm) were used for trapping. A terra cotta pot was chosen as the container for all the tests because it was identified as the best container for termite trapping in preliminary station trials [21]. The volume of the container was chosen as the unit of measurement; each terra cotta pots filled with substrate was moistened with 100 mL of water. The substrates were obtained from farmers and herders in the village of Nasso. The pots were ordered from a potter of Bobo-Dioulasso. The experimental device used was a completely randomized block design (blocks dispersed over time) of 6 treatments and 4 replicates per day for 5 days where the blocks were assimilated to successive essays [17,18,19,20]. All harvests were carried out at 7 a.m., i.e., 24 h after the traps were set. Twenty-four 2-dm^3^ terra cotta pots were used each day and each treatment was repeated four times a day for five days. Five successive trials (blocks) (5 days) were carried out every two months from February 2018 to December 2018. A total of 30 essays were carried out during the experiment. In addition to these variables, after weighting all termites, for all replicates, soldiers were sorted out and weighted separately.

### 2.7. Influence of Sites on Termite Trapping

In Nasso and in the forest of Dinderesso, essays were carried out in the dry season (April and December) and in the rainy season (August) to evaluate the influence of sites in different seasons on termite trapping. Using maize stems as substrate, a test was conducted simultaneously at both sites. For 4 days, every morning (at 7 a.m.), 5 terra cotta pots of 2 dm^3^ containing maize stems moistened with 100 mL of water each were placed at the two sites. Harvesting took place the next day at the same time when other terra cotta pots were placed. The experimental design was a completely randomized block design (blocks dispersed over time) of 2 treatments (trapping site) and 5 replicates where the blocks were assimilated to successive essays [17,18,19,20]. A total of 15 essays were carried out during the experiment.

### 2.8. Influence of the Volume of Containers on Termite Trapping

To assess the effect of the volume of the containers on the quantity of termites harvested, three terra cotta pots of different volumes (2 dm^3^, 4 dm^3^, and 6 dm^3^) and opening diameters (16 cm, 20 cm, and 24 cm, respectively) were used with maize stems to trap termites. To humidify the substrate, 100 mL, 200 mL, and 300 mL of water were used respectively for the three volumes of terra cotta pots. A completely random block system, dispersed over the time of three treatments (container volume: 2 dm^3^, 4 dm^3^, and 6 dm^3^) and 2 replicates was used. Ten successive trials (lasting 10 days) were carried out in the months of April, August, and December 2019. A total of 30 essays were carried out during the experiment.

### 2.9. Influence of Trapping Duration on Termite Trapping

Using 2-dm^3^ terra cotta pots with a 16-cm diameter opening and maize stems as substrate, the effect of the duration of trap deposition on the quantity of termites harvested was assessed at the Nasso station. Each essay lasted three days. Each day at 7 a.m. on three successive days, 10 terra cotta pots of 2 dm^3^ filled with maize stems moistened with 100 mL of water were placed on pre-identified termite tracks. On the fourth day, at 7 a.m., all 30 terra cotta pots were collected (10 terra cotta pots had stayed on the soil during 24 h, 10 terra cotta pots for 48 h, and 10 terra cotta pots for 72 h). From that day on and for the next three days, the same process was repeated a second time. A system of completely random blocks, dispersed over the time of three treatments (trap settling time: 24 h, 48 h, and 72 h) and 10 replicates was carried out. Two successive trials (7 days) were carried out every two months, from April 2019 to February 2020. A total of 12 essays were carried out during the experiment.

### 2.10. Influence of Harvest Time on Termite Trapping

The essays carried out during this experiment were aimed at identifying the best time for termite harvesting according to the season (dry and rainy). To do this, 2-dm^3^ terra cotta pots with a 16-cm diameter opening were used with maize stems to trap termites. One hundred mL of water per terra cotta pot was used to moisten the substrate. The traps were placed and harvested at the following times of the day: 6 a.m., 8 a.m., 10 a.m., 12 a.m., 2 p.m., 4 p.m., and 6 p.m. All traps were left 24 h, thus, traps deposited at 6 a.m. were collected the next day at 6 a.m. and so on. The experimental design was a completely randomized block (blocks scattered over time) of 7 treatments (harvest hours) and 5 repetitions per day where the blocks were assimilated to successive trials. Four successive trials (4 days) were carried out every two months from April 2019 to February 2020. A total of 24 essays were carried out during the experiment.

### 2.11. Statistical Analysis

The statistical analyses were carried out using R software version 3.6.0 [22]. Analyses of the effect of the volume of containers (2, 4 and 6 dm^3^) were conducted on the weight of dry termites produced per dm^3^. Analyses on the effect of duration of exposure (24, 48, and 72 h) were conducted on the weight of dry termites produced per 24 h. The normality of the data and the homogeneity of the variance were tested using the Shapiro–Wilk test and the Fligner test. Based on these, statistical tests were selected for the comparisons of means. To assess the effect of the type of containers on the weight of the termites harvested, the Kruskall–Wallis test was used. To assess the influence of substrates, seasons, sites, volume of container, and harvesting hours, one-way and two-way ANOVAs (with a month as the second fixed factor) were performed. The comparison of the means was carried out with the Student–Newman–Keuls test or the Tukey test at the 5% level.

## 3. Results

### 3.1. Influence of Containers on Termite Trapping

**Figure 4:**
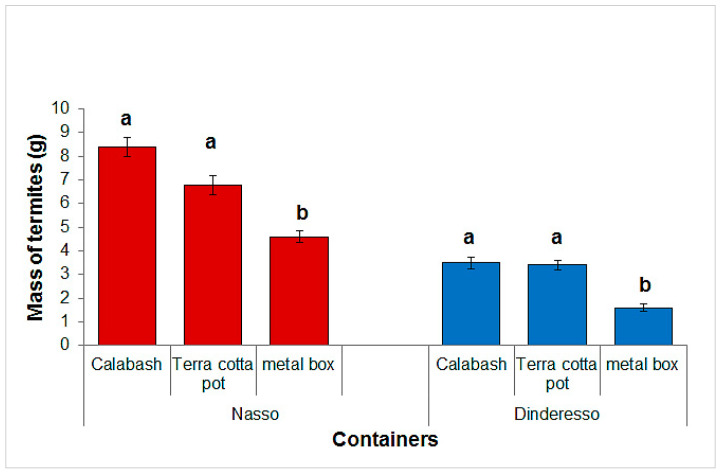
Average masses of dry termites harvested per container, according to container’s type and site. The histograms represent the averages of 20 replicates and those with the same letters at the same site are not significantly different at the 5% level. Error bars represent standard errors.

Figure 4 shows the quantities of termites ranging from 4.6 g to 8.4 g in Nasso and from 1.6 g to 3.5 g in Dinderesso. There was a significant difference between the quantity of termites collected in the different containers in Nasso (chi-squared = 13.165; df = 2, 57; *p* < 0.001) and in Dinderesso (chi-squared = 16.043; df = 2, 57; *p* < 0.001), with the metal box collecting less termites than the two other containers types at both sites.

### 3.2. Influence of Seasons and Substrates on Termite Trapping

**Figure 5:**
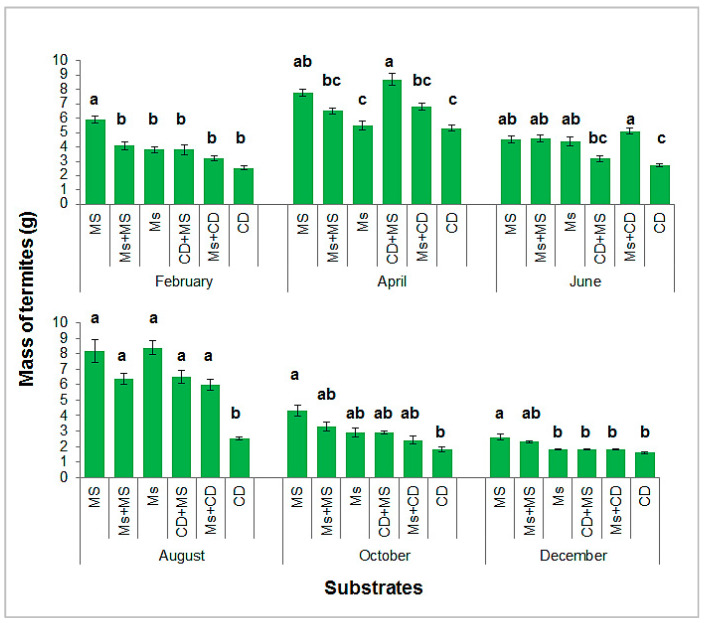
Average masses of dry termites harvested per container according to the substrates (MS = maize stems; Ms + MS = maize cobs + maize stems; Ms = maize cobs; CD + MS = cow dung + maize stems; and Ms + CD = maize cobs + cow dung; CD = cow dung). Error bars represent standard errors. Histograms with the same letter, in the same month, are not significantly different at the 5% level.

Both the substrate (F = 17.990; df = 5, 684; *p* < 0.0001) and the month of trapping (F = 79.964; df = 5, 684; *p* < 0.0001) as well as interactions (F = 3.285; df = 25, 684; *p* < 0.0001) were significant in a 2-way ANOVA. Figure 5 shows the quantities of termites harvested based on six treatments in six months of the year. The highest quantities were obtained from maize stems in February (5.92 g); the combination of maize stems and cow dung in April (8.7 g); the combination of maize cobs and cow dung in June (5.1 g); maize cobs in August (8.4 g); maize stems in October (4.3 g); and maize stems in December (2.6 g). For all months, the lowest average was obtained by the cow dung substrate. Masses for this substrate varied from 1.6 g in December to 5.3 g in August. Highly significant differences were found between substrates in February (F = 5.217; df = 5, 114; *p* < 0.0001), April (F = 5.983; df = 5, 114; *p* < 0.0001), and August (F = 7.672; df = 5, 114; *p* < 0.0001); significant differences in June (F = 4.066; df = 5, 114; *p* < 0.01), December (F = 4.194; df = 5, 114; *p* < 0.01), and October (F = 2.948; df = 5, 114; *p* < 0.05). The months of April and August were the most productive and the month of December the least productive.

**Figure 6:**
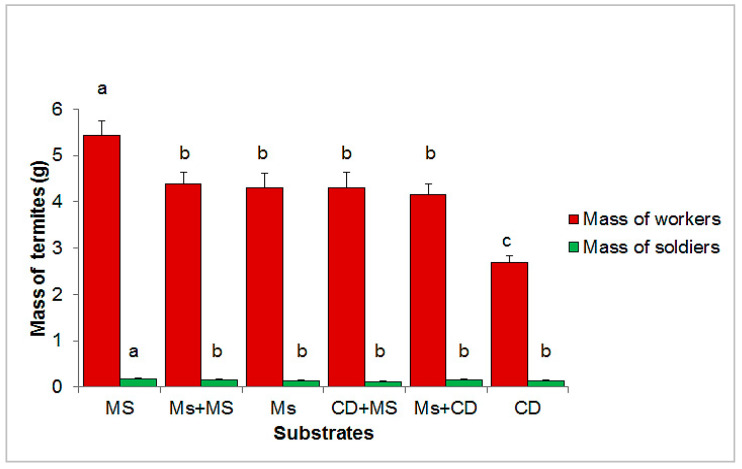
Average masses of soldiers and workers harvested per container according to substrates (MS = maize stems; Ms + Ms = maize cobs + maize stems; Ms = maize cobs; CD + MS = cow dung + maize stems; and Ms + CD = maize cobs + cow dung; CD = cow dung). Error bars represent standard errors. Histograms with the same letter are not significantly different at the 5% level. Means ± SE for soldiers: MS: 0.181 ± 0.012; Ms + MS: 0.146 ± 0.010; Ms: 0.143 ± 0.010; CD + MS: 0.119 ± 0.008; Ms + CD: 0.147 ± 0.010; and CD: 0.143 ± 0.009.

Figure 6 shows the average masses of workers and soldiers harvested according to the substrate of all months counted together. The quantity of termites (soldiers + workers) ranged from 2.8 g from cow dung to 5.6 g from maize stems. Statistical analyses showed a significant difference in the total amount of harvested termites (workers: F = 10.96; df = 5, 714; *p* < 0.0001; soldiers: F = 4.06; df = 5, 714; *p* = 0.001) among substrates. However, in all substrates, soldiers’ weight represented a very small part of the samples, from 2.7% (cow dung + maize stem) to 5.2% (cow dung).

### 3.3. Influence of Sites on Termite Trapping

**Figure 7:**
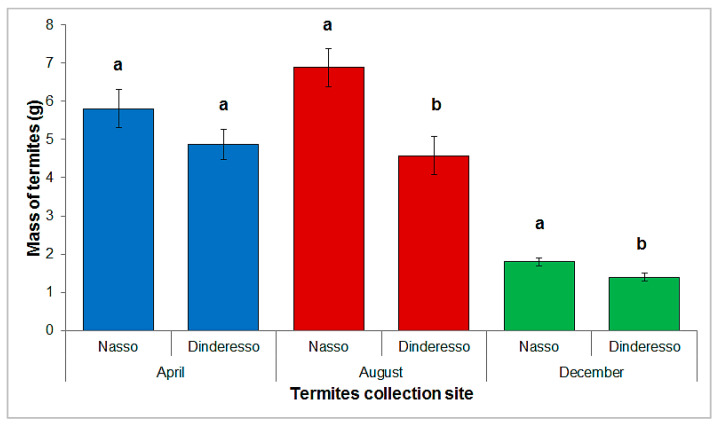
Average masses of termites harvested per container according to sites and months. Histograms with the same letter in the same month are not significantly different at the 5% level. Error bars represent standard errors.

Both the site (F = 14.542; df = 1, 114; *p* = 0.0002) and the month of trapping (F = 94.655; df = 2, 114; *p* < 0.0001) as well as interactions (F = 4.285; df = 2, 114; *p* = 0.016) were significant in 2-way ANOVA. Figure 7 shows the average masses of termites harvested according to the sites in the rainy season (August), the hot dry season (April), and the cold dry season (December). For each period, the quantities collected in Nasso were higher than those collected in Dinderesso. Statistical analyses showed significant differences between the termite masses collected at the two sites in August (F = 20.315; df = 1, 38; *p* < 0.0001) and December (F = 2.486; df = 1, 38; *p* < 0.01). In April, there was no significant difference between sites (F = 0.319; df = 1, 38; *p* = 0.7).

### 3.4. Influence of the Volume of Containers on Termite Trapping

**Figure 8:**
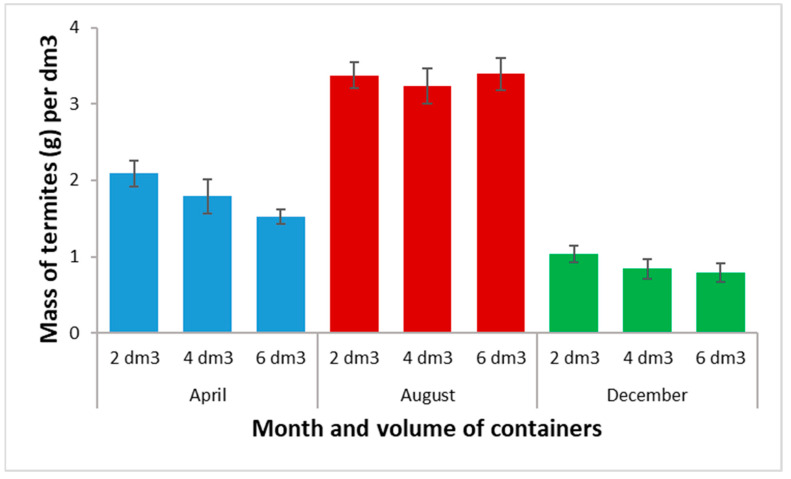
Average masses of termites harvested per container and per dm^3^ of volume, according to the volume of containers. In the same month, there was no significant difference between containers’ volumes at the 5% level. Error bars represent standard errors.

Figure 8 shows the average quantities of termites harvested per dm^3^ of volume, according to the volume of the containers. Bigger containers provided higher amounts of termites, which varied from 2.07 g to 6.74 g for the 2-dm^3^ terra cotta; from 3.36 g to 12.95 g for the 4-dm^3^ terra cotta pots; and from 4.72 g to 20.35 g for the 6-dm^3^ terra cotta. However, there was no significant difference in the mass of termite harvested per dm^3^ among containers’ volumes (F = 2.02; df = 2, 171; *p* = 0.136). In contrast, the month of trapping was highly significant (F = 158.14; df = 2, 171; *p* < 0.0001). The best yields were obtained in August regardless of the volume of the terra cotta pots and the lowest averages were obtained in December.

### 3.5. Influence of Trapping Duration on Termite Trapping

**Figure 9:**
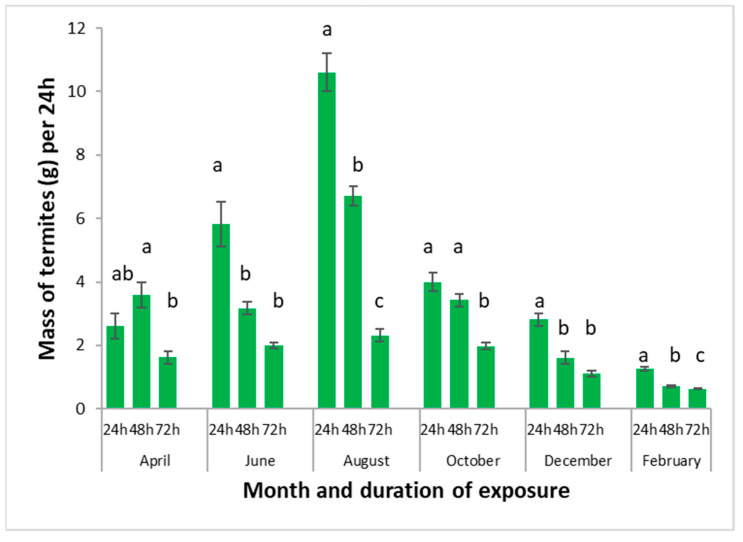
Average masses of termites harvested per container and per 24 h, according to trapping duration. Error bars represent standard errors. Histograms with the same letter in the same month are not significantly different at the 5% level.

Figure 9 shows the quantities of termites harvested per 24 h of exposure, according to the length of time the traps were set. Both the duration (F = 158.99; df = 2, 342; *p* < 0.0001) and month of trapping (F = 141.49; df = 5, 342; *p* < 0.0001) as well as interactions (F = 27.15; df = 10, 342; *p* < 0.0001) were significant in a 2-way ANOVA. The highest quantities per 24 h were obtained 24 h after deposition in all months except in April, and the third day always provided a lower number of termites than the first two days. The highest yields were obtained in August and the lowest ones in February.

### 3.6. Study of the Influence of Harvest Hours on Termite Trapping

**Figure 10:**
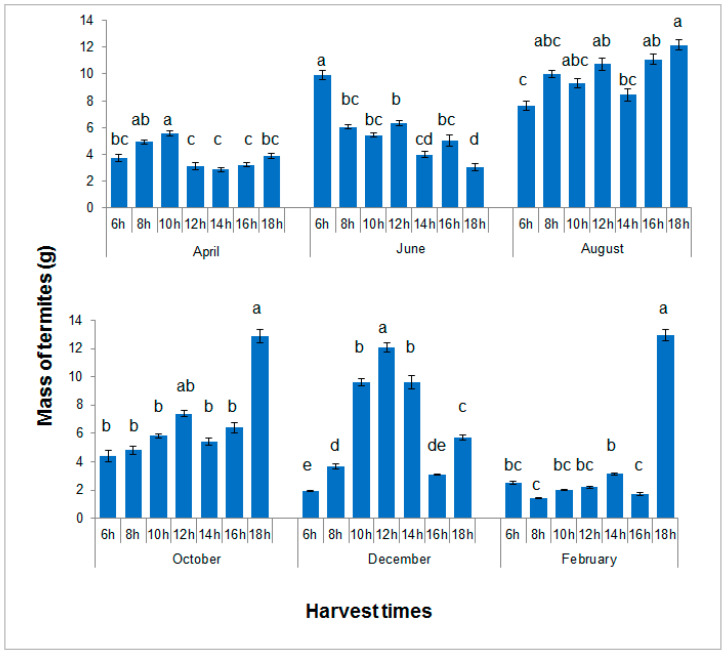
Average masses of termites harvested per container at different hours of the day. Histograms with the same letter during the same month are not significantly different at the 5% level. Error bars represent standard errors.

In all months, significant differences in yields were found among harvest hours: April (F = 6.248; df = 6, 133; *p* < 0.0001), June (F = 15.169; df = 6, 133; *p* < 0.0001), August (F = 4.335; df = 6, 133; *p* < 0.0001), October (F = 20.180; df = 6, 133; *p* < 0.0001), December (F = 62,129; df = 6, 133; *p* < 0.0001), and February (F = 134.650; df = 6, 133; *p* < 0.0001) (Figure 10). However, the best time for harvesting was highly variable among seasons. In February and October, the highest yields were obtained at 6pm, in June at 6am, and in December at midday. The highest average was obtained in February at 6 pm (12.97 g) and the lowest also in February at 8 a.m. (2.52 g). Average temperature and relative humidity at harvest are shown in Supplementary File S1, Table S2. In December and February, the two months showing the highest variations in yield among harvest times, the most successful harvest occurred when humidity was lowest and the least successful when the temperature was lowest. In other months, which showed less variations in temperature and humidity during the day, yields were less variable.

## 4. Discussion

The tests carried out in this study have enabled us to assess factors that affect the trapping success of termites of the genus *Macrotermes* in Burkina Faso. The study of the effect of the containers showed that terra cotta pots and calabashes are more effective than iron boxes for trapping *Macrotermes* spp., despite the fact that iron boxes (e.g., large tomato cans) are commonly used for that purpose in Burkina Faso [8]. The effectiveness of the terra cotta container could be explained by its composition. Terra cotta pots are porous, allowing excess moisture to evaporate, and at the same time, insulating the substrate from extreme temperatures. Their effectiveness for trapping termites had already been mentioned by Farina et al. and Ouedraogo [23,24]. Recently, Dao et al. [14] also observed that terra cotta pots were more effective to collect *Macrotermes* spp. and *Odontotermes* spp. than iron boxes and plastic buckets. The effectiveness of calabash could be explained by the fact that it itself constitutes a substrate in addition to the food baits that are introduced to attract termites. Its use in Burkina Faso for termite trapping was cited by Van Huis [25]. However, in terms of durability, the terra cotta pots appear to be the best container for *Macrotermes* spp. trapping because, after a few days of use, the calabash is fully destroyed by termites.

The use of terra cotta pots of different sizes during three different months (April, August, and December) shows that the quantity of termites harvested is proportional to the volume of the terra cotta pot, at least within the range of the volumes tested in this study. Thus, the larger the pot, the greater the quantity of termites harvested.

In addition to the containers, termite trapping success also varies with the substrate. For *Macrotermes* spp., maize residues, in particular maize cobs, give better results than cow dung and mixtures including cow dung. This is due to a preference of termite species for certain feed sources over others [26]. *Macrotermes* spp., being fungus-growing termites, harvest a wide range of plant material with which they build a fungus comb in termite mounds, which degrades the substrates of the plants harvested by the termites [1,27,28,29]. Among our tested substrates, cow dung is the least effective substrate to trap *Macrotermes* spp. in any season of the year. This could be explained by the fact that cow dung, although being a substrate of plant origin, has already undergone decomposition through its passage in the digestive tract of cattle. This could make it less attractive to termites, seeming to prefer the residues of undecomposed maize. Substrates, however, have to be selected according to the termite species targeted. Recent experiments made in Northern Burkina Faso [14] showed that *Macrotermes* spp. are preferably attracted by sorghum stems and much less by cow dung and mixtures containing cow dung, as observed in this study. In contrast, cow dung and mixtures containing cow dung were very effective to trap species of the genus *Odontotermes*.

For all substrates tested, the number of soldiers harvested was very small compared to the high number of workers (from 2.7% to 5.2% in weight but much less in numbers because soldiers are much heavier than workers). The role of workers in the termite mound mainly consists of harvesting and transporting plant debris from outside to the termite mound [30]. Thus, they are easily lured and trapped in the containers full of attractive substrates. Soldiers, whose role is to defend the colony [31], are found in small quantities in the traps. This is fortunate because *Macrotermes* soldiers are known to hurt and even kill young chicks by biting their throat and, therefore, it is usually advised to give *Macrotermes* spp. only to poultry older than a month [7,8]. A solution to avoid injuries would be to dry the termites, as done in our studies, but the extraction of termites from the substrate would be too time-consuming for the farmer. It is likely that methods involving the collection of termites directly from mounds may result in a higher proportion of soldiers, even though farmers have developed techniques to avoid the collection of a high quantity of soldiers even when termites are collected from the mounds [30].

Our trials carried out in all seasons showed that the trapping of *Macrotermes* spp. can be conducted throughout the year. However, there are variations in the quantities of termites harvested according to the seasons. In general, the lowest harvests were obtained in December (cold dry season), and the highest in the middle of the rainy season in August. The hot dry season in April was also more favorable than the cold dry season in December. This could be due to the seasonal variations in temperature and relative humidity (Supplementary File S1, Tables S1 and S2). The activity of *Macrotermes* spp. is known to be very sensitive to climatic conditions [1,32]. In Northern Burkina Faso, Dao et al. [14] also collected more *Macrotermes* spp. in the rainy season than in the dry season. Interestingly, in surveys in both Burkina Faso [8] and Ghana [7], farmers tended to say that trapping with baited inverted containers is particularly well adapted to the dry season since, in the rainy season, termite tracks are usually washed off by the rain. This comment, however, is probably more applicable for *Odontotermes* spp., whose tracks and mounds are less conspicuous than those of *Macrotermes* spp.

It must be noted that the experiment on the influence of the season and substrates may have been biased by the fact that the placement and harvest of termite traps was done at 7 a.m. whereas another experiment showed that the most favorable time for harvest dramatically changes with seasons. Even during months that, at first view, seem less favorable for *Macrotermes* spp. trapping, sizeable amounts can be obtained by choosing the right time of the day for harvesting. For example, the month of February recorded the highest mass when the harvest took place at 6 pm. In December, when temperatures are the lowest, harvesting at midday in full sunshine provided the best results. These observations disagree with the results of surveys of poultry farmers in Burkina Faso by Diawara [13] and Dao et al. [8] who stated that, to get the maximum number of termites, the harvest had to be done before sunrise. For the trapping of *Macrotermes* spp., this statement was true only for the month of June, at the beginning of the rainy season. For the other months, especially in the cold dry season, our results show that it is quite the opposite. It is commonly stated that termites hide during the day and are most active at night [26,33]. The fact that large quantities of termites are collected at different times of the day could be explained by the terra cotta’s thermoregulatory capacities, which could favor the termites’ daytime activity. In addition, the terra cotta pots do not allow daylight to pass, which could also be favorable for termite activity. Termites of the genus *Macrotermes* are known to be extraordinary adaptive [26], and we can therefore hypothesize that the termites shift their period of activity from night to day to ensure the transport of nutrients from the outside into the termite mound when climatic conditions are favorable.

Tests carried out in Nasso and Dinderesso to evaluate the influence of habitats on the trapping of *Macrotermes* spp. showed that more termites were caught in Nasso (savannah) than in Dinderesso (classified forest), throughout the year. The difference was greater in the dry season (April and December) than in the rainy season (August). This was surprising considering that the Dinderesso site seemed to be richer in termites, with much larger *Macrotermes* mounds than the Nasso site. This could be explained by the biomass cover of the soil. Indeed, the forest soil in Dinderesso is entirely covered by plant debris and dead leaves (Figure 2), so that the termites have enough food at their disposal and therefore were probably less interested in the substrate that was brought to them. At Nasso, despite the absence of large termite mounds, the presence of a soil poorly covered by dead plants may have favored a better colonization of the substrates, hence the better termite harvest at this site compared to Dinderesso.

Another experiment that tested the number of termites trapped after one, two, and three days showed that the highest number of termites trapped per 24 h was obtained after one day and the lowest after three days. Thus, if enough substrate is available, it is recommended to empty the traps and renew the substrate on a daily basis. However, this experiment was conducted with small 2-dm^3^ pots, in which the substrate was quickly consumed. It is possible that larger pots containing higher amounts of substrate may be kept longer.

Finally, it is important to note that, in contrast to other termite collection methods based on mounds destruction or on digging holes in the mounds e.g., [8,30]. The trapping method with reversed pots used in this study could contribute significantly to the protection of termite mounds and to the preservation of biodiversity in general. Indeed, according to farmers, this trapping technique allows them to collect close to the farm over a long period without affecting termite populations.

## 5. Conclusions

The development of local protein resources such as termites, which are low-cost, suitable, and can be used by all poultry farmers, requires the sustainable optimization of harvesting techniques for these resources. This study has enabled us to provide information to farmers on how to optimize the trapping of *Macrotermes* species. The various tests have shown that the quantity of termites harvested varies according to the season, the containers, the substrates, the habitat, the length of time the traps have been set, and the time of harvest. Taking each factor into account can considerably improve the quantity of termites trapped and their use as a protein source in poultry feed among smallholder poultry farmers. These data are appropriate for *Macrotermes* but preliminary data suggest that other genera suitable for trapping such as *Odontotermes* react differently. It would be important for breeders to be able to use several species of termites during all seasons of the year. To diversify the sources of protein available to improve traditional poultry farming in West Africa, studies on other species such as *Odontotermes* spp. are needed.

## Data Availability

All data of this study are publicly available in Supplementary File S2.

## Supplementary Materials

The following are available online at https://www.mdpi.com/article/10.3390/insects13010062/s1, Supplementary File S1. Figure S1: Termite sorting process; Table S1: Average temperature and relative humidity during termite trapping tests; Table S2: Average temperature and relative humidity during the study of the influence of harvest hours on termite trapping. Supplementary File S2: Rough data of all experiments.
