# Supplementary material for: Sustainable Use of Macrotermes spp. to Improve Traditional Poultry Farming through an Efficient Trapping System in Burkina Faso
Source: Insects. 2022 Jan 5;13(1):62. doi: 10.3390/insects13010062 (PMC8779955; doi:10.3390/insects13010062)
Supplement: Supplementary file 1 [file insects-13-00062-s001.zip › Supplementary file S1.pdf]

Supplementary File 1

Sustainable use of *Macrotermes* spp. to improve traditional poultry farming through an efficient trapping system in Burkina Faso

Aïchatou Nadia Christelle Dao, Fernand Sankara, Salimata Pousga, Kalifa Coulibaly, Jacques Philippe Nacoulma, Irénée Somda, Marc Kenis

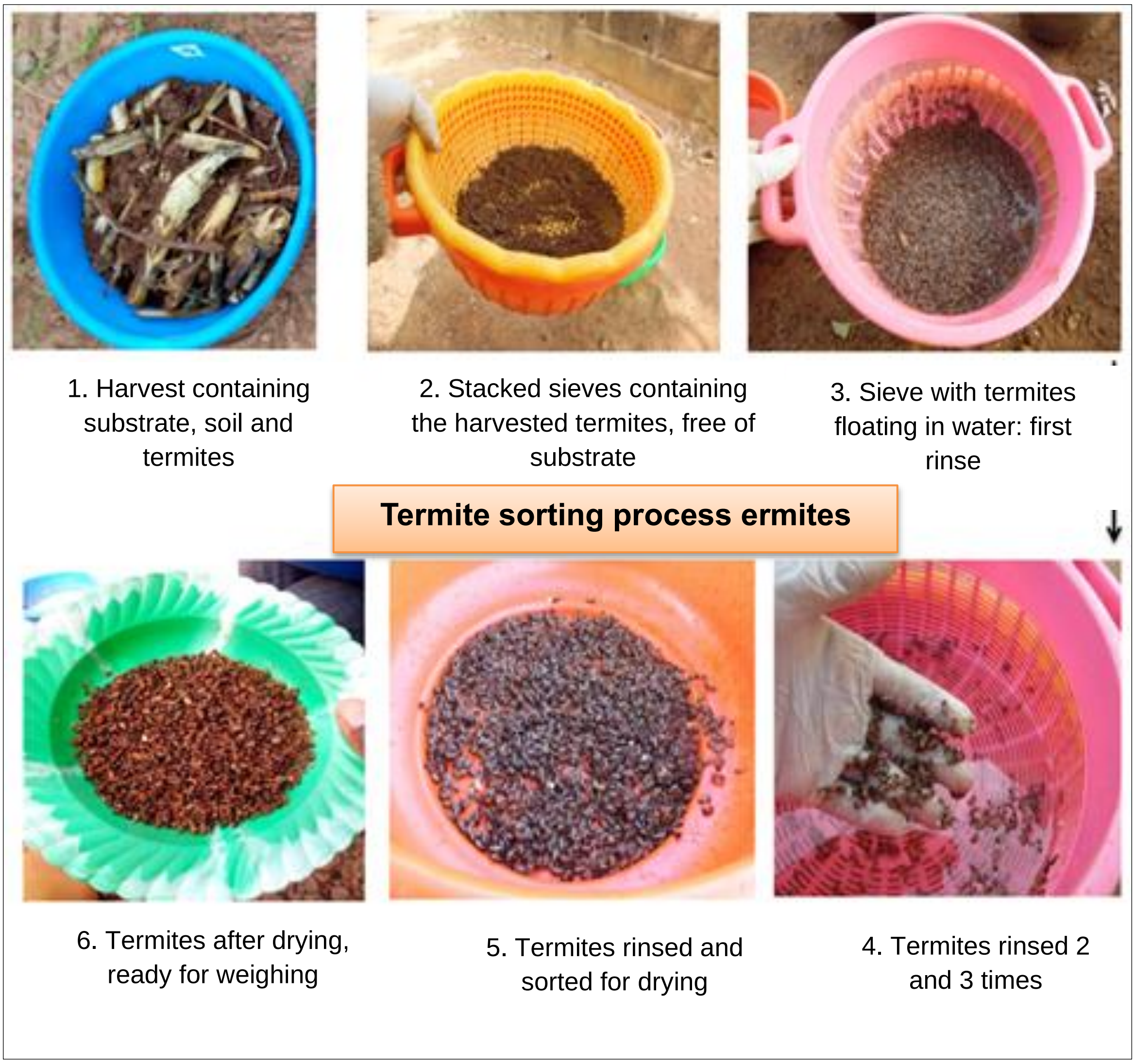

Figure S1: Termite sorting process

Table S1: Average temperature and relative humidity during the termite trapping tests

Months	Test of substrates		Test of sites				Test of volume of containers		Test of trapping duration	
	Average temperature at harvest (°C)	Average humidity at harvest (%)	Average temperature at harvest (°C) at Nasso	Average humidity at harvest (%) at Nasso	Average temperature at harvest (°C) at Dinderesso	Average humidity at harvest (%) at Dinderesso	Average temperature at harvest (°C)	Average humidity at harvest (%)	Average temperature at harvest (°C)	Average humidity at harvest (%)
February	26.0	45.2	-	-	-	-	-	-	16.0	35.0
April	27.1	67.8	28.5	56.5	29.8	54.9	27.7	37.2	27.5	72.0
June	23.4	92.6	-	-	-	-	-	-	20.6	84.7
August	24.2	94.9	24.7	91.9	23.0	82.2	24.4	91.9	24.5	91.2
October	23.4	86.7	-	-	-	-	-	-	25.4	83.0
December	14.6	67.6	15	65.3	13.2	70.5	16.3	63.4	15	68.2

Table S2: Average temperature and relative humidity during the study of the influence of harvest hours on termite trapping.

	6h		8h		10h		12h		14h		16h		18h	
	Average temperature at harvest (°C)	Average humidity at harvest (%)	Average temperature at harvest (°C)	Average humidity at harvest (%)	Average temperature at harvest (°C)	Average humidity at harvest (%)	Average temperature at harvest (°C)	Average humidity at harvest (%)	Average temperature at harvest (°C)	Average humidity at harvest (%)	Average temperature at harvest (°C)	Average humidity at harvest (%)	Average temperature at harvest (°C)	Average humidity at harvest (%)
February	14.5	50.5	20.5	40.6	28.3	13.6	34.0	13.6	35.7	13.1	35.9	13.2	30.5	14.4
April	22.6	63.0	27.8	51.5	31.7	27.7	36.0	21.6	37.5	17.0	37.6	14.6	34.0	25.1
June	23.4	92.0	25.8	87.2	28.8	74.5	30.1	70.1	30.1	69.1	30.5	69.2	28.4	78.1
August	23.7	94.2	25.4	96.4	28.0	79.1	28.7	73.6	29.4	73.1	27.6	88.1	26.3	88.9
October	21.8	86.5	24.2	89.6	27.9	75.2	32.3	49.6	33.0	45.5	31.9	46.7	29.0	68.9
December	14.7	66.0	16.6	67.6	24.3	27.1	29.0	17.9	32.3	13.2	31.1	14.6	25.5	43.5
